# Changes in concentrations of haemostatic and inflammatory biomarkers in synovial fluid after intra-articular injection of lipopolysaccharide in horses

**DOI:** 10.1186/s12917-017-1089-1

**Published:** 2017-06-19

**Authors:** Stine Mandrup Andreassen, Anne Mette Lindberg Vinther, Søren Saxmose Nielsen, Pia Haubro Andersen, Aziz Tnibar, Annemarie T. Kristensen, Stine Jacobsen

**Affiliations:** 1 Department of Veterinary Clinical Sciences, Section of Large Animal Medicine and Surgery, University of Copenhagen, Højbakkegård Allé 5, DK-2630 Tåstrup, Denmark; 2 Department of Veterinary Clinical Sciences, University of Copenhagen, Dyrlægevej 16, DK-1870 Frederiksberg C, Denmark; 3 Department of Veterinary and Animal Sciences, University of Copenhagen, Grønnegårdsvej 8, DK-1870 Frederiksberg C, Denmark; 4 Department of Clinical Sciences, Swedish Agricultural University, 75007 Uppsala, Sweden

**Keywords:** Equine, Acute arthritis, Haemostasis, Inflammation, Thrombin-antithrombin, d-dimer, Fibrinogen, Haptoglobin, Iron, Serum amyloid A

## Abstract

**Background:**

Septic arthritis is a common and potentially devastating disease characterized by severe intra-articular (IA) inflammation and fibrin deposition. Research into equine joint pathologies has focused on inflammation, but recent research in humans suggests that both haemostatic and inflammatory pathways are activated in the joint compartment in arthritic conditions. The aim of this study was to characterize the IA haemostatic and inflammatory responses in horses with experimental lipopolysaccharide (LPS)-induced joint inflammation. Inflammation was induced by IA injection of LPS into one antebrachiocarpal joint of six horses. Horses were evaluated clinically with subjective grading of lameness, and blood and synovial fluid (SF) samples were collected at post injection hours (PIH) -120, −96, −24, 0, 2, 4, 8, 16, 24, 36, 48, 72 and 144. Total protein (TP), white blood cell counts (WBC), serum amyloid A (SAA), haptoglobin, iron, fibrinogen, thrombin-antithrombin (TAT) and d-dimer concentrations were assessed in blood and SF.

**Results:**

Intra-articular injection of LPS caused local and systemic signs of inflammation including increased rectal temperature, lameness and increased joint circumference and skin temperature. Most of the biomarkers (TP, WBC, haptoglobin, fibrinogen and TAT) measured in SF increased quickly after LPS injection (at PIH 2–4), whereas SAA and d-dimer levels increased more slowly (at PIH 16 and 144, respectively). SF iron concentrations did not change statistically significantly. Blood WBC, SAA, haptoglobin and fibrinogen increased and iron decreased significantly in response to the IA LPS injection, while TAT and d-dimer concentrations did not change. Repeated pre-injection arthrocenteses caused significant changes in SF concentrations of TP, WBC and haptoglobin.

**Conclusion:**

Similar to inflammatory joint disease in humans, joint inflammation in horses was accompanied by an IA haemostatic response with changes in fibrinogen, TAT and d-dimer concentrations. Inflammatory and haemostatic responses were induced simultaneously and may likely interact. Further studies of interactions between the two responses are needed for a better understanding of pathogenesis of joint disease in horses. Knowledge of effects of repeated arthrocenteses on levels of SF biomarkers may be of value when markers are used for diagnostic purposes.

## Background

Septic arthritis is a common and potentially devastating disease affecting horses. The chance of full athletic recovery has been reported to range from 81 to as low as 25% [1–4], even where aggressive treatment regimens are used. To better understand prognostic factors and investigate novel approaches to diagnosis and effective management of inflammatory joint conditions in horses, the pathogenesis of intra-articular (IA) disease needs to be further elucidated.

Research into the pathogenesis of arthritis in horses has focused on inflammation in IA tissues, diagnostic assessment of inflammatory markers such as white blood cell counts (WBC), total protein (TP), serum amyloid A (SAA), pro-inflammatory cytokines, and eicosanoids in synovial fluid (SF), and on measures to control inflammation [5–9]. Less attention has been given to the causes and effects of fibrin in the inflamed joint; and the possible interaction between haemostasis and inflammation, which is well-described in human joint disease [10, 11], has not been investigated in horses. Increased concentrations of thrombin-antithrombin (TAT) complexes have been demonstrated in SF from humans with rheumatoid arthritis (RA) [11], which shows that thrombin activation and coagulation takes place in the inflamed joint compartment. Inflamed human SF has been shown to contain increased levels of haemostatic proteins such as thrombin [12] and antithrombin [13], both of which have been shown to attract leukocytes to the joint and to enhance IA inflammation [11, 12, 14]. Also fibrinogen may play a role in the pathogenesis of IA inflammation [15, 16]. In non-inflamed SF from humans fibrinogen was absent or found in very low levels [13, 17], and in inflammatory joint conditions, such as RA, osteoarthritis (OA) and antigen-induced arthritis, increased SF fibrinogen concentration has been demonstrated [17–19]. Fibrinogen is a key protein in haemostasis, interacting with platelets to form a fibrin-platelet clot to control haemorrhage or exudation from the circulation. Fibrin/fibrinogen has also been shown to induce inflammatory reactions in human and equine synoviocytes [20, 21], and fibrinogen may thus contribute to both haemostasis and inflammation in the joint compartment. Fibrinogen is also an acute phase reactant, and similar to other acute phase reactants, such as SAA, haptoglobin and iron, plasma concentrations of fibrinogen will change in response to inflammatory and infectious conditions [22].

In human RA, which is characterised by severe IA inflammation, fibrin deposition takes place inside the joint [19, 23]. This has led to the suggestion that in arthritic conditions there is a dysbalance between fibrinogenesis and fibrinolysis [24], and that an overall state of hypercoagulability is at play [24]. It has been suggested that fibrinolysis is essential for complete resolution of inflammatory joint diseases [11, 19]. For assessment of IA fibrinolysis, d-dimer, a break-down product released during fibrinolysis of cross-linked fibrin, may prove useful. D-dimer is present in equine SF, and higher concentrations were detected in SF of foals with septic arthritis and in SF of horses with osteochondritis dissecans (OCD) than in SF from healthy controls [25, 26].

The aim of the study was to describe the IA haemostatic response in an equine experimental model of inflammatory arthritis. Several factors involved in haemostasis (fibrinogen, TAT, d-dimer) and inflammatory biomarkers (WBC, TP, SAA, haptoglobin, iron) were assessed sequentially after IA injection of lipopolysaccharide (LPS). It was hypothesized that IA haemostasis would be active concomitantly with the acute inflammation.

## Methods

### Horses

Six research horses, 3 Danish Warmblood and 3 mixed breed horses, 3 mares and 3 geldings aged 3–14 years and weighing 425–620 kg, were included in the study. The horses were included if they were free of clinical signs of inflammation, and levels of inflammatory parameters (WBC and differential leukocyte count, SAA, iron, fibrinogen) in blood were within reference ranges. Horses were included if lameness assessment including lunging, and palpation and flexion tests of the carpal joints were unremarkable, and if SF levels of WBC, differential leukocyte count, and TP concentrations in the antebrachiocarpal joint were within normal limits. One horse showed a positive response to distal limb flexion indicating fetlock pathology; the horse was included, as it had no response to carpal flexion, normal findings on palpation of the carpus, and normal parameters in SF from the antebrachiocarpal joint. This horse was excluded from analyses of lameness scores. The included horses participated in a larger open experimental crossover study involving intravenous and IA injection of LPS. Only data relating to the IA administration is presented, the results from the intravenous LPS injection are presented in the paper by Vinther et al. (2016) [27]. Horses were housed in box stalls, had free access to hay and water and were fed twice daily with a commercial grain mixture.

### Study design

Two randomly selected horses (horse E, F) received LPS IA as their first treatment, while 4 horses (horses A, B, C, D) had been subject to systemic experimental inflammation induced by intravenous injection of LPS four weeks prior to the IA LPS injection. The four-week washout was selected to eliminate possible effects of LPS tolerance [28, 29]. Before the study was initiated, all horses were trained with positive reinforcement to reduce the need for sedation during experimental procedures such as arthrocentesis, in order to minimize potential analgesic and anti-inflammatory effects of sedatives. On three to five occasions horses (B, D, E) needed sedation with xylazine (0.2 mg/kg, Narcoxyl® vet, MSD Animal Health, Denmark) to obtain an SF sample of sufficient volume.

### Induction of joint inflammation

Joint inflammation was induced in one antebrachiocarpal joint by injection of 3 μg LPS derived from *Escherichia coli* strain 055:B5 (# L2880, Sigma-Aldrich Denmark ApS) as described previously [30, 31].

Aliquots of LPS, stored in siliconized glass tubes at −20 °C, were thawed and vortexed approximately 30 min to break down micelle formation, diluted to a final concentration of 3 μg LPS/ml in Ringers Acetate® (Baxter A/S, Allerød, Denmark). The final LPS solution was vortexed again for 30 min and 1 ml was transferred to a syringe and immediately injected using aseptic technique. The injection time was defined as post injection hour (PIH) 0.

### Experimental procedures

Blood and SF samples from the injected antebrachiocarpal joint were obtained before IA injection of LPS at PIH -120; −96; −24, and 0 to allow assessment of potential effects of repeated arthrocenteses on concentrations of SF biomarkers [32]. After LPS injection, blood and SF from the injected joint was sampled at PIH 2, 4, 8, 16, 24, 36, 48, 72, and 144.

At each sampling point the horses underwent a clinical examination with assessment of general appearance, respiratory frequency, rectal temperature, and heart rate. Pain assessment and lameness scoring using the AAEP lameness scale (the scale ranges from 0 to 5, with 0 being no perceptible lameness, and 5 being most extreme with little or no weight bearing) [33] was performed by two observers (SMA, AMLV) blinded to each other’s grading. Pain was assessed by the previously described composite measure pain scale [34], which is based on six behavioural categories: gross pain behaviour, weight bearing, head position relative to the withers, location in stall, response to open door, and response to approach from the observer, as well as on an ‘overall’ subjective pain score. Point scores for all behavioural categories were totalled to yield a final pain score ranging from 0 to 23. The injected antebrachiocarpal joint was subjectively evaluated by palpation for heat, pain and swelling. Moreover, the skin temperature of the dorsal clipped carpus was assessed by infrared thermometry at a distance of 20 cm according to the manufacturer’s recommendation (Raytek Raynger MX4, Raytek, Santa Cruz, California) and reported as the mean of 5 measurements. Joint circumference was measured with a tape measure at the level of the accessory carpal bone.

### Samples and analyses

Blood was collected through an indwelling jugular venous catheter and immediately transferred to tubes in the following order: serum, citrate and ethylenediaminetetraacetic acid (EDTA) in accordance with instructions from the manufacturer (BD Vacutainer®, BD A/S, Albertslund, Denmark). The first five mL of blood were discarded. The catheter was flushed with saline; no heparin was used in the study. Three to 10 mL of SF was aspirated aseptically from the injected antebrachiocarpal joint with a 21 gauge 40 mm long needle and immediately transferred to 2.7 ml tubes (BD vacutainer®) containing 3.2% buffered sodium citrate (1.5 mL of SF was added to each tube to obtain a citrate:SF ratio equal to the theoretic citrate:plasma ratio estimated at 45%^1^) and to a 4 mL-, spray-coated EDTA tube (BD vacutainer®, approximately 2 .2 ml of SF added to each tube^2^). All tubes were inverted carefully 5–10 times directly after sampling. At each sampling point, SF was assessed macroscopically (colour, viscosity, and transparency). Inflammatory (WBC, TP, SAA, haptoglobin, iron) and haemostatic (fibrinogen, TAT, d-dimer) biomarkers were measured in fresh and stored blood/plasma/serum and SF as detailed in Table 1.

**Table 1:** Information on measured biomarkers in blood and synovial fluid samples incl. Sample treatment, storage and laboratory analyses

Parameter	Sample	Tubes	Centrifugation	Storage	Time to analysis	Assay	References
White blood cell count	Blood	EDTA		2–8 °C	Max. 24 h	Automated cell counting, ADVIA 2120 analyzer (Siemens Healthcare Diagnostics Inc., Deerfield, Illinois, USA)	[31, 78]
	Synovial fluid	EDTA		2–8 °C	Max. 24 h	Counted in haemocytometer	[31, 78]
Total protein	Blood	Serum	1200 g for 10 min at 20 °C	2–8 °C	Max. 24 h	Biuret, ADVIA 1800 Chemistry System (Siemens Healthcare Diagnostics Inc., Deerfield, Illinois, USA)	[36]
	Synovial fluid	EDTA		Max. 1 h at room temperature	Within 1 h	Refractometry	[31, 78]
Serum amyloid A	Blood	Serum	1200 g for 10 min at 20 °C	2–8 °C	Max. 24 h	Immunoturbidometry (LZ test SAA, EIKEN Chemical Co., Tokyo, Japan)	[79]
	Synovial fluid	EDTA		Max. 24 h at 2–8 °C, then in aliquots at −80 °C	Within 7 months		[30]
Haptoglobin	Blood	Serum	1200 g for 10 min at 20 °C	Max. 4 h at room temperature then in aliquots at −80 °C	Within 7 months	Biochemical peroxidase assay in duplicates (Phase Range Hp Assay, Tridelta Development Ltd. Kildare, Ireland)	[60]
	Synovial fluid	EDTA		Max. 24 h at 2–8 °C, then in aliquots at −80 °C	Within 7 months		Previously determined in porcine synovial fluid by ELISA [80]
Iron	Blood	Serum	1200 g for 10 min at 20 °C	2–8 °C	Max. 24 h	Colorimetric spectrophotometry (ADVIA 1650, Bayer A/S, Lyngby, Denmark).	[37, 78]
	Synovial fluid	EDTA		Max. 24 h at 2–8 °C, then in aliquots at −80 °C	Within 7 months		Not previously reported in equine synovial fluid, but in synovial fluid of human patients by a colorimetric method using an automatic analyser (Aeroset, USA) [70]
Fibrinogen	Blood	Citrate		2–8 °C	Max. 24 h	Clauss method, automated coagulometric analyser (ACL 9000, Instrumentation Laboratory, Barcelona, Spain).	[22]
	Synovial fluid	Citrate	1500 g for 15 min at 20 °C	Initially 15–30 min at room temperature then in aliquots at −80 °C	Within 17 months	QuickVet® Equine Fibrinogen™ Test (Scandinavian Micro Biodevices, Farum, Denmark)	Measured in synovial fluid in human osteoarthritis with automatic coagulometer (BFT II Dade Behring, Marburg, Germany) [17]
Thrombin-antithrombin	Blood	Citrate	1500 g for 15 min at 20 °C	Initially 15–30 min at room temperature then in aliquots at −80 °C	Within 20 months	ELISA measured in duplicates (Enzygnost TAT micro test kit, Siemens Healthcare Diagnostics, Denmark)	[45, 81]
	Synovial fluid	Citrate			Within 20 months		Not previously reported in equine synovial fluid, but with same assay in synovial fluid of human rheumatoid arthritis and osteoarthritis [14]
D-dimer	Blood	Citrate	1500 g for 15 min at 20 °C	Initially 15–30 min at room temperature then in aliquots at −80 °C	Within 21 months	Immunoturbidimetry in duplicates (STA-Liatest D-DI, Diagnostica Stago, Triolab, Denmark)	[82, 83]
	Synovial fluid	Citrate			Within 18 months		[25, 26], both used a quantitative immune-turbidimetric latex agglutination assay (Miniquant, Biopool, Trinity Biotech, Wicklow, Ireland)

*EDTA* Ethylenediaminetetraacetic acid

*ELISA* Enzyme-linked immunosorbent assay

Fibrinogen was measured in SF using the QuickVet® Equine Fibrinogen™ Test (Scandinavian Micro Biodevices, Farum, Denmark). This assay has not previously been used with SF, and its suitability for measuring fibrinogen in SF was therefore validated. Imprecision was assessed by repeated measurements over two days on 10 machines with one cartridge (from the same batch) per measurement to obtain the maximum possible variations. Pooled samples with high (2.06 ± 0.1 g/L), intermediate (0.4 ± 0.13 g/L) and low (0.27 ± 0.13 g/L) fibrinogen concentrations were made from SF samples from 6, 5 and 2 horses, respectively, and used for the analyses. The overall imprecision (coefficient of variation) was 4.68% for the high pool (18 repeats), 22.5% for the intermediate pool (19 repeats), and 32.0% for the low pool (10 repeats). For the intermediate and low pools, results (one measurement in each pool) with a recorded fibrinogen concentration of 0 g/L were omitted from the calculations, as they were interpreted as deficient aspiration of viscous sample into the cartridge. Inclusion of these measurements increased the coefficients of variation to 32.6% and 47.4% for intermediate and low pools, respectively. Inaccuracy was assessed by linearity under dilution. Triplicate determinations of fibrinogen concentrations were made using a synovial pool with high concentrations of fibrinogen diluted 0, 10, 20, 30, 40, 50, 60, 70, 80, 90 and 100% using a synovial pool with low fibrinogen content. Linear regression showed that the slope did not deviate from 1 (slope = 0.996, 95% confidence interval = 0.86–1) and the intercept did not deviate from 0. Runs test, however, revealed that data deviated from a linear model (*P* = 0.024). Our validation of the fibrinogen assay thus showed slight inaccuracy and large imprecision in the low concentration range. Whether this imprecision was related to the high viscosity of SF was not clear. Currently, the assay is thus mainly relevant for monitoring of fibrinogen concentrations in the high concentrations range, for detection of substantial changes in SF fibrinogen concentrations or for sequential assessment of SF fibrinogen in the same individual.

### Data analyses

Each of the outcome parameters, except lameness score, was compared to the first pre-injection value (at PIH = −120) using a random intercept, random slope model in R [35]. The outcome parameters were transformed to achieve residuals (ε), which were deemed independent identically distributed Normal (0, σ^2^) except for fibrinogen in SF, rectal temperature and carpus circumference, which did not need to be transformed. The general model used was thus:

$$ \mathrm{transformation}\left(\mathrm{outcome}\ \mathrm{parameter}\right)=\upalpha +\mathrm{time}+\mathrm{time}\ \mathrm{x}\ \mathrm{horse}+\upvarepsilon $$

where: transformation(outcome parameter), was the transformed outcome parameter for a specific outcome parameter; time, was the fixed and random effect of a specific time-point within a horse. Time was included as a categorical parameter. The results were presented as pairwise comparisons between first pre-injection time (PIH = −120) and subsequent time points.

Imprecision (coefficients of variance) and inaccuracy (linear regression analysis and runs test) calculations for fibrinogen measurements in SF were done in Microsoft Excel (Microsoft Office Professional Plus 2010 Microsoft Corporation) and GraphPad Prism 5.0 (GraphPad Software, Inc., CA, USA). Statistical significance was defined as *P* < 0.05 for all analyses.

## Results

**Fig. 1:**
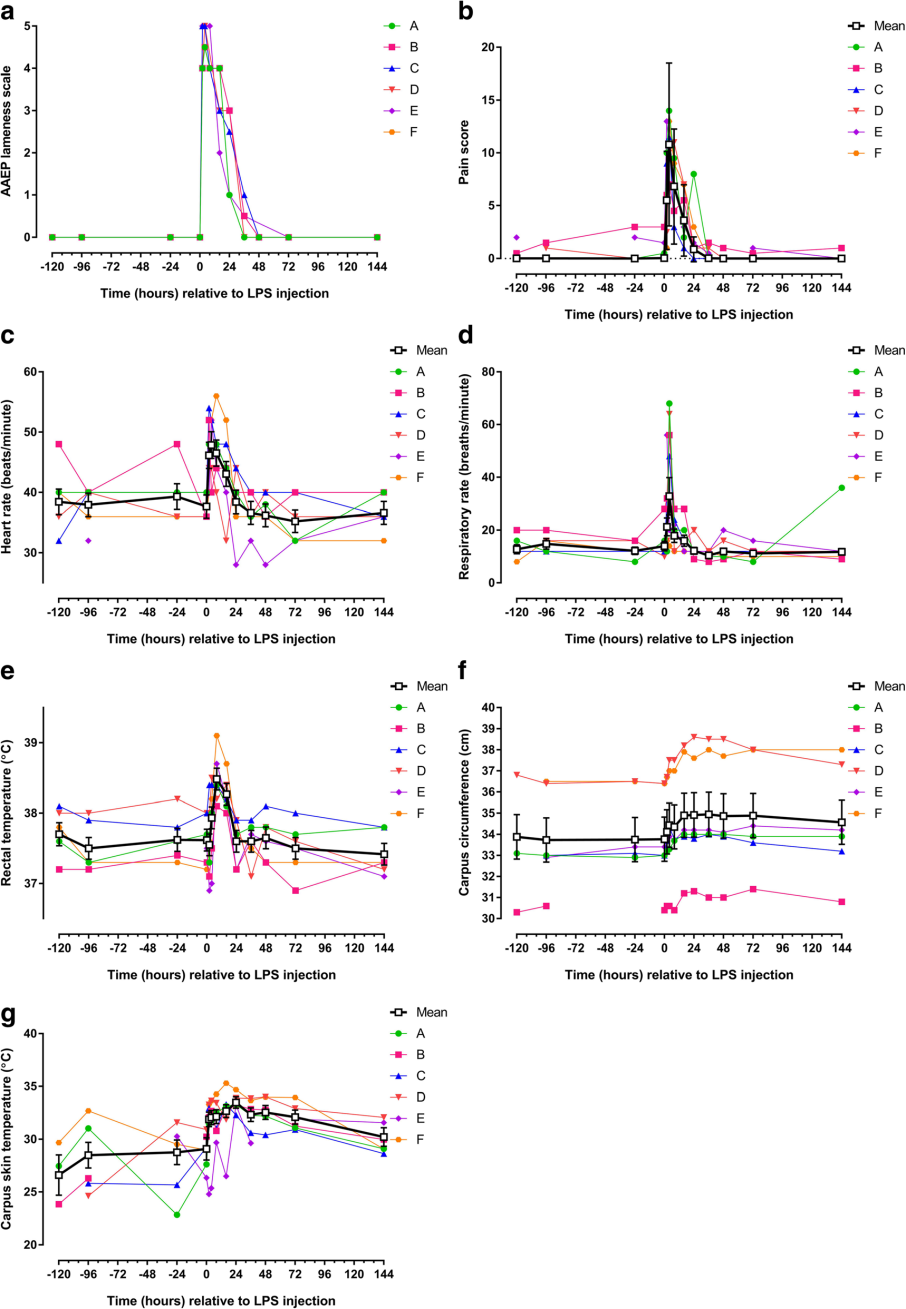
Clinical parameters recorded in 6 horses before and after intra-articular injection of 3 μg *E. coli* O55:B5 lipopolysaccharide (LPS) in one antebrachiocarpal joint (**a**) Association of American Equine Practitioners [AAEP] lameness scale; **b** composite measure pain score; **c** heart rate; **d** respiratory rate; **e** rectal temperature; **f** circumference of injected carpus in centimetres [cm]; **g** skin temperature over injected carpus). Individual horse responses and estimated means ± standard error of the mean (*black line*) are depicted. Results of the statistical analyses are shown in Table 2

Two different comparisons were made to describe changes in clinical parameters and biomarker concentrations: post-injection parameters (PIH 2–144) were compared to PIH −120 to assess LPS-induced changes in biomarker concentrations; and pre-injection parameters (PIH -96; −24; 0) were compared to PIH −120 to assess effects of repeated arthrocenteses on biomarker concentrations. In the following, results are summarized in Tables 2 (clinical parameters), 3 (biomarkers in blood), and 4 (biomarkers in SF) and shown in Fig. 1 (clinical parameters), 2 (inflammatory biomarkers in blood), 3 (haemostatic biomarkers in blood), 4 (inflammatory biomarkers in SF), and 5 (haemostatic biomarkers in SF).

**Table 2:** Clinical parameters (estimated means and ranges) before and after intra-articular injection of 3 μg *E. coli* O55:B5 lipopolysaccharide

	Pain score (Fig. 1b)		Heart rate (Fig. 1c)		Respiratory rate (Fig. 1d)		Rectal temperature (Fig. 1e)		Carpus circumference (Fig. 1f)		Carpus skin temperature (Fig. 1g)	
			Beats/min		Breaths/min		°C		Centimetres		°C	
Hours relative to LPS injection	Estimated mean	Range	Estimated mean	Range	Estimated mean	Range	Estimated mean	Range	Estimated mean	Range	Estimated mean	Range
−120	0.02	0–2	38.4	32–48	12.8	8–20	37.7	37.2–38.1	33.9	30.3–36.8	26.6	23.84–29.68
−96	0.02	0–1.5	38.0	32–40	14.8	12–20	37.5	37.2–38.0	33.7	30.6–36.5	28.5	24.62–32.70
−24	0.01	0–3	39.3	36–48	12.2	8–16	37.6	37.3–38.2	33.8	32.9–36.5	28.8	22.84–31.60
0	0.04	0–3	37.6	36–40	13.9	10–28	37.6	37.2–38.0	33.8	30.4–36.4	29.1	26.34–31.60
2	5.50**	1–13	46.1	40–54	21.3	12–56	37.6	36.9–38.4	34.1	30.6–36.7	31.9*	24.80–30.92
4	10.80***	7–14	47.9*	40–52	32.8*	14–68	37.9	37.0–38.5	34.4*	30.6–37.5	32.1*	25.36–33.66
8	6.82**	3–11	46.5	40–56	17.9	12–28	38.5**	38.1–39.1	34.4	30.4–37.5	32.2**	29.68–34.28
16	3.60*	1–7	43.0	32–52	16.0	12–28	38.3*	38.0–38.7	34.9***	31.2–38.2	32.7***	26.50–35.30
24	0.88	0–8	38.4	28–44	12.2	9–20	37.6	37.2–37.9	34.9***	31.3–38.6	33.5***	32.30–34.68
36	0.03	0–1.5	36.6	32–40	10.5	8–12	37.6	37.1–37.9	35.0***	31.0–38.5	32.3**	29.64–33.88
48	0	0–1	36.2	28–40	11.9	9–20	37.7	37.3–38.1	34.9***	31.0–38.5	32.6**	30.42–34.02
72	0	0–1	35.2	32–40	11.2	8–16	37.5	36.9–38.0	34.9***	31.4–38.0	32.1**	30.92–33.96
144	0	0–1	36.6	32–40	11.8	9–36	37.4	37.1–37.8	34.6***	30.8–38.0	30.2	28.64–32.08

Asterisks designate significant difference from post-injection hour −120 (* = *P* < 0.05; ** = *P* < 0.01 and *** = *P* < 0.001)

*LPS* lipopolysaccharide

### Pre-injection period: effects of repeated arthrocenteses

**Fig. 2:**
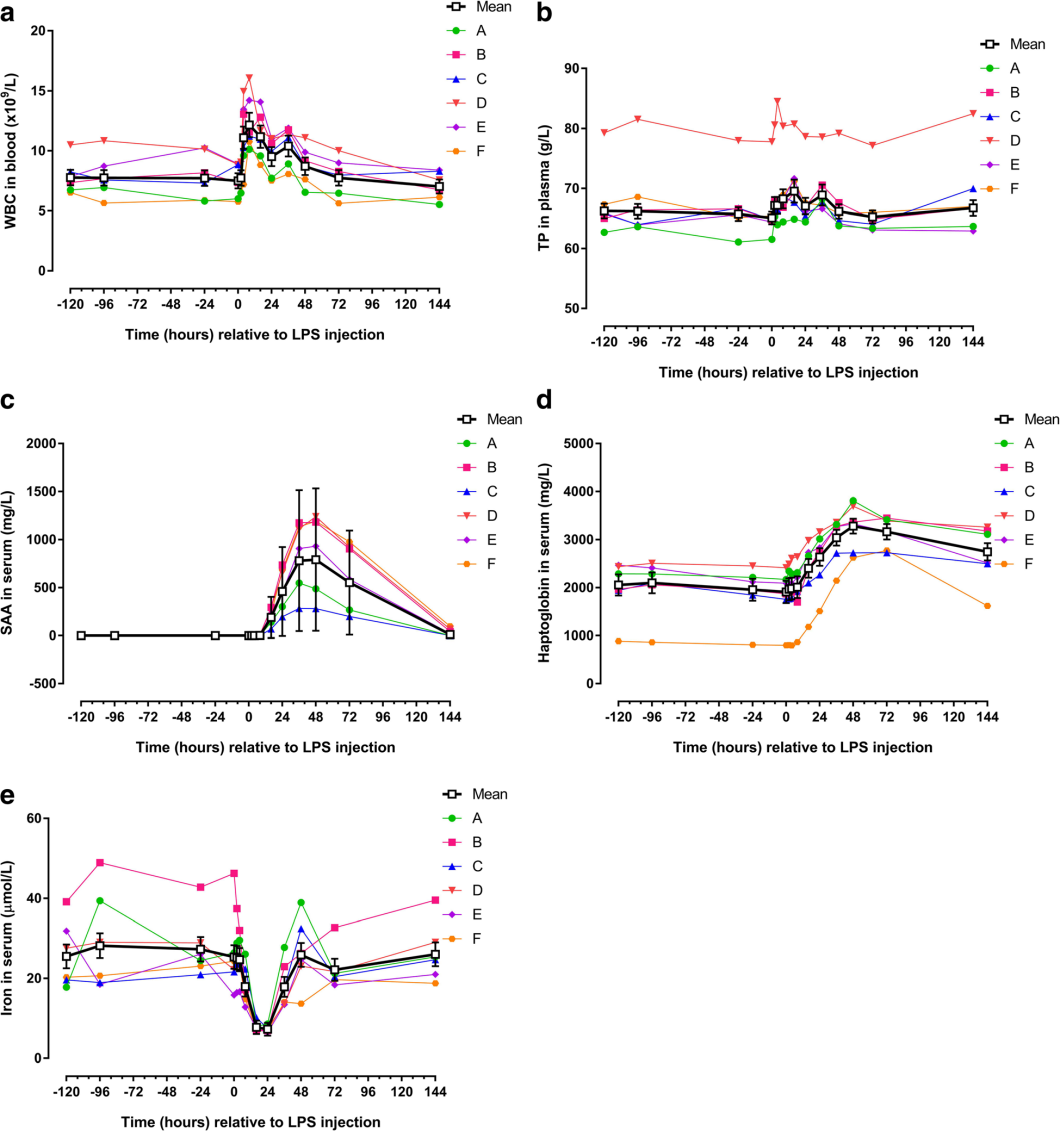
Concentrations/levels of inflammatory biomarkers measured in blood from 6 horses before and after intra-articular injection of 3 μg *E. coli* O55:B5 lipopolysaccharide (LPS) in one antebrachiocarpal joint (**a** white blood cell count [WBC]; **b** total protein [TP]; **c** serum amyloid A [SAA]; **d** haptoglobin; **e** iron). Individual horse responses and estimated means ± standard error of the mean (*black line*) are depicted. Results of the statistical analyses are shown in Table 3

**Fig. 3:**
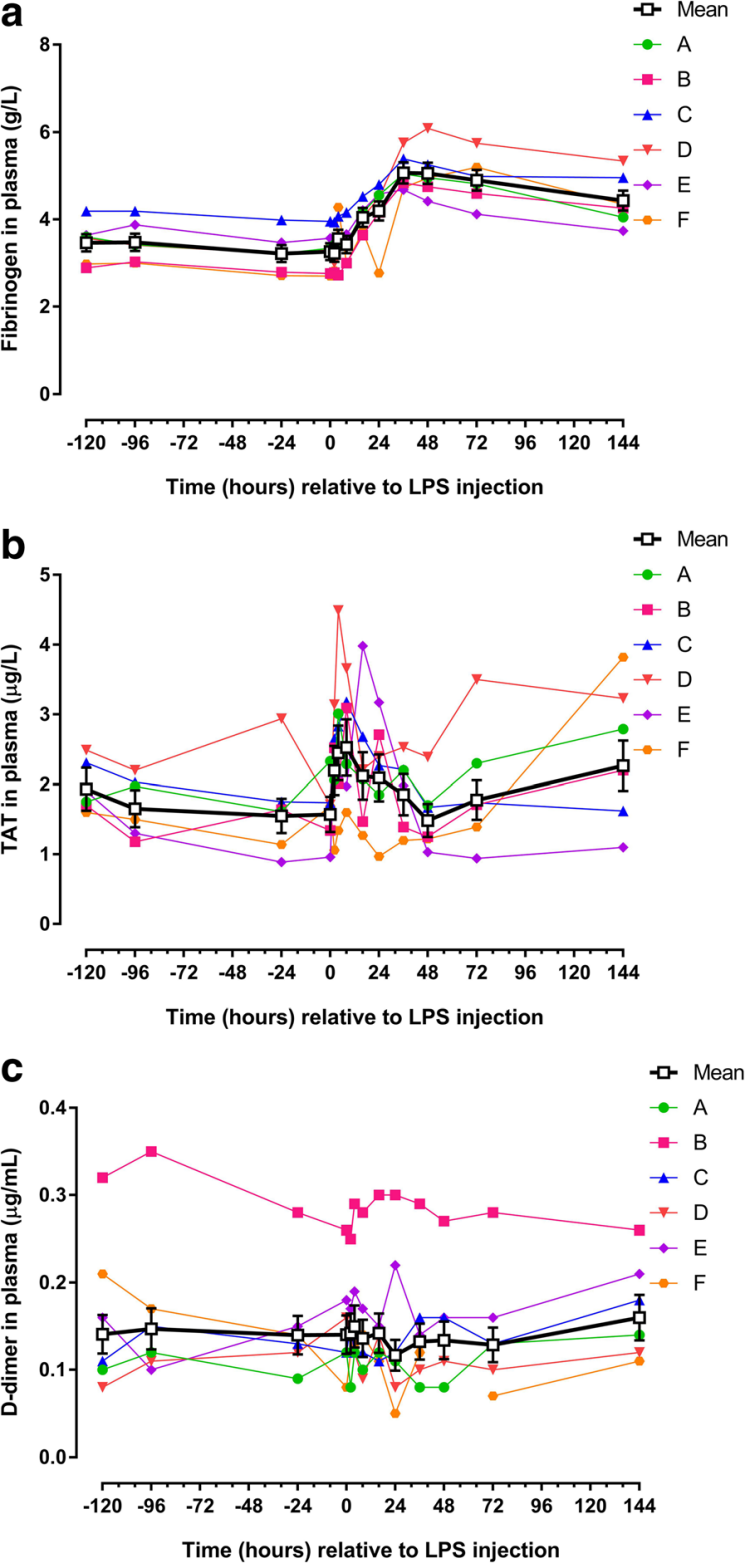
Concentrations/levels of haemostatic biomarkers measured in blood from 6 horses before and after intra-articular injection of 3 μg *E. coli* O55:B5 lipopolysaccharide (LPS) in one antebrachiocarpal joint (**a** fibrinogen; **b** thrombin-antithrombin [TAT]; **c** d-dimer). Individual horse responses and estimated means ± standard error of the mean (*black line*) are depicted. Results of the statistical analyses are shown in Table 3

**Fig. 4:**
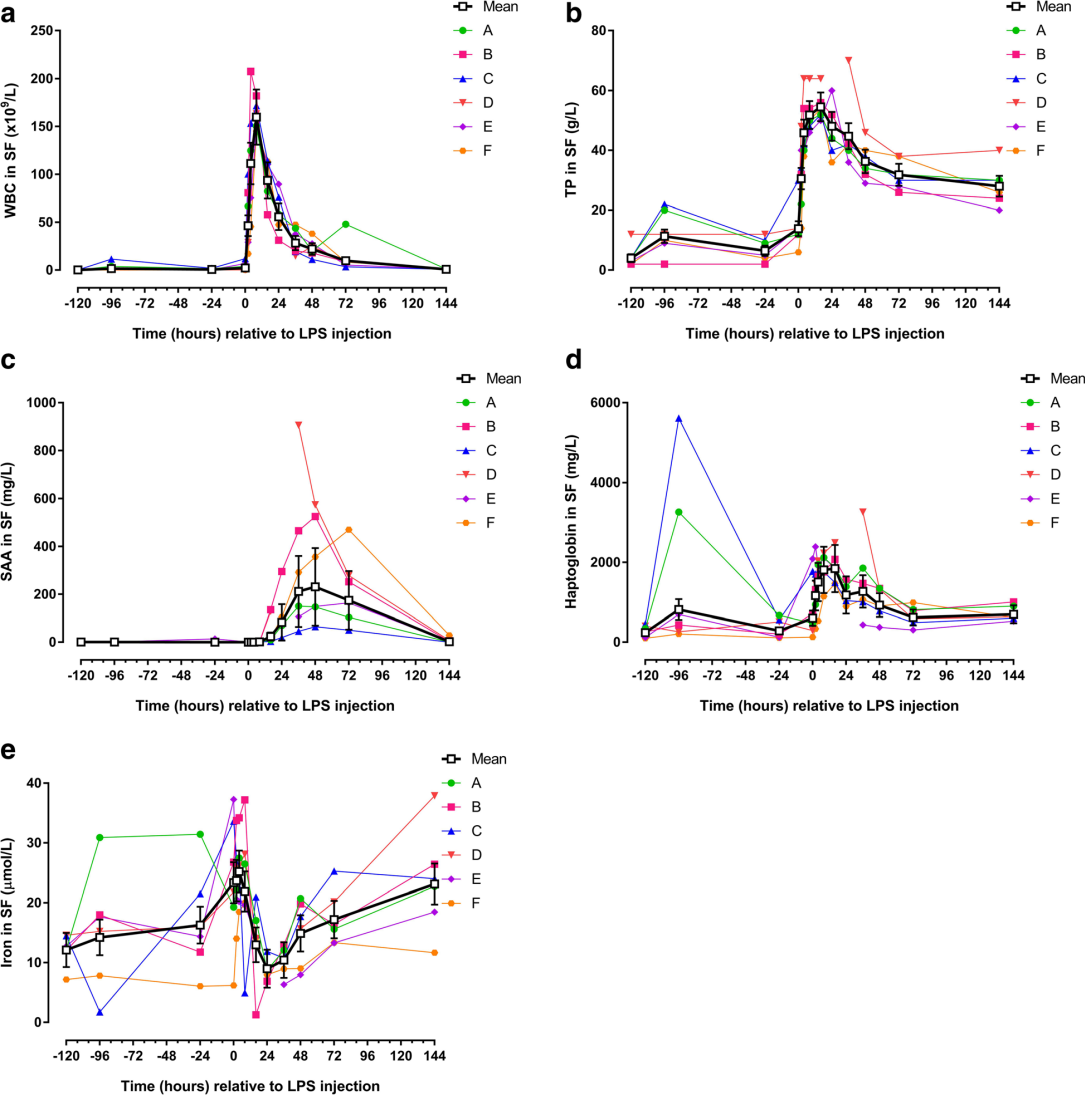
Concentrations/levels of inflammatory biomarkers measured in synovial fluid (SF) from 6 horses before and after intra-articular injection of 3 μg *E. coli* O55:B5 lipopolysaccharide (LPS) in one antebrachiocarpal joint (**a** white blood cell count [WBC]; **b** total protein [TP]; **c** serum amyloid A [SAA]; **d** haptoglobin; **e** iron). Individual horse responses and estimated means ± standard error of the mean (black line) are depicted. Results of the statistical analyses are shown in Table 4

There were no significant changes in clinical parameters or blood biomarkers in the pre-injection period (Figs. 1a-g, 2a-e and 3a-c; Tables 2 and 3). SF WBC (Fig. 4a), TP (Fig. 4b) and haptoglobin (Fig. 4d) increased on one or two occasions in response to previous arthrocentesis in the pre-injection period. These increases were statistically significant for WBC at PIH 0 (*P* < 0.05), TP at PIH −96 and 0 (*P* < 0.05 and *P* < 0.001, respectively); and haptoglobin at PIH -96 (*P* < 0.05) (Table 4).

**Table 3:** Blood biomarkers (estimated means and ranges) before and after intra-articular injection of 3 μg *E. coli* O55:B5 lipopolysaccharide

	White blood cell count (Fig. 2a)		Total protein (Fig. 2b)		Serum amyloid A (Fig. 2c)		Haptoglobin (Fig. 2d)		Iron (Fig. 2e)		Fibrinogen (Fig. 3a)		Thrombin-antithrombin (Fig. 3b)		D-dimer (Fig. 3c)	
	× 10^9^/L		g/L		mg/L		mg/L		μmol/L		g/L		μg/L		μg/mL	
Hours relative to LPS injection	Estimated mean	Range	Estimated mean	Range	Estimated mean	Range	Estimated mean	Range	Estimated mean	Range	Estimated mean	Range	Estimated mean	Range	Estimated mean	Range
−120	7.8	6.5–10.5	66.3	62.7–79.3	< DL	< DL	2056.1	886.8–2466.5	25.5	17.8–39.1	3.46	2.89–4.19	1.9	1.6–2.5	0.14	0.08–0.32
−96	7.7	5.7–10.9	66.2	63.7–81.5	< DL	< DL- 1.2	2097.3	864.8–2509.0	28.2	18.5–48.9	3.48	3.00–4.19	1.7	1.2–2.2	0.15	0.10–0.35
−24	7.7	5.8–10.3	65.7	61.1–78.0	< DL	< DL	1955.0	812.3–2451.5	27.3	20.9–42.8	3.22	2.71–3.99	1.5	0.9–2.9	0.14	0.09–0.28
0	7.5	5.8–8.9	65.1	61.6–77.8	< DL	< DL	1908.2	802.6–2414.2	25.4	15.8–46.3	3.26	2.70–3.96	1.6	1.0–2.3	0.14	0.08–0.26
2	7.7	6.5–9.1	67.2	64.5–80.6	< DL	< DL	1964.2	806.4–2488.5	25.2	16.5–37.4	3.22	2.78–3.94	2.2	1.1–3.1	0.14	0.08–0.25
4	11.1***	7.2–15.0	67.2	64.0–84.5	< DL	< DL	1980.0	799.7–2609.2	24.7	16.6–32.0	3.56	2.72–4.28	2.4	1.3–4.5	0.15	0.12–0.29
8	12.2***	10.1–16.1	68.3	64.5–80.4	2.2	0.8–8.0	2009.3	868.4–2642.5	17.9	12.8–26.1	3.43	2.99–4.16	2.5	1.6–3.7	0.14	0.09–0.28
16	11.2***	8.8–14.1	69.6	64.9–80.8	189.0***	69.0–292.6	2403.8*	1180.2–2982.0	7.8***	6.8–10.1	4.05	3.64–4.53	2.1	1.3–4.0	0.14	0.11–0.30
24	9.5***	7.5–11.0	67.1	64.5–78.7	459.3***	195.8–735.5	2636.6***	1507.5–3161.3	7.3***	6.4–8.6	4.20*	2.77–4.80	2.1	1.0–3.2	0.12	0.05–0.30
36	10.4***	8.1–11.9	66.2	66.6–78.6	780.4***	282.8–1176.4	3041.3***	2143.8–3359.8	17.9	13.5–27.8	5.07***	4.68–5.76	1.9	1.2–2.5	0.13	0.08–0.29
48	8.7	6.6–11.1	66.2	63.8–79.2	791.3***	281.5–1240.3	3284.3***	2626.4–3813.8	25.9	13.7–38.9	5.06***	4.42–6.09	1.5	1.0–2.4	0.13	0.08–0.27
72	7.8	5.6–10.0	65.3	63.1–77.2	552.4***	198.7–976.9	3166.3***	2727.6–3451.3	22.1	18.4–32.7	4.90***	4.12–5.75	1.8	0.9–3.5	0.13	0.07–0.28
144	7.0	5.5–8.4	66.8	63.0–82.5	10.5**	<DL-96.8	2746.6***	1617.1–3261.0	26.0	18.8–39.5	4.43**	3.74–5.34	2.3	1.1–3.8	0.16	0.11–0.26

Asterisks designate significant difference from post-injection hour −120 (* = *P* < 0.05; ** = *P* < 0.01 and *** = *P* < 0.001)

*DL* Detection limit

*LPS* Lipopolysaccharide

**Table 4:** Synovial fluid biomarkers (estimated means and ranges) before and after intra-articular injection of 3 μg *E. coli* O55:B5 lipopolysaccharide

	White blood cell count (Fig. 4a)		Total protein (Fig. 4b)		Serum amyloid A (Fig. 4c)		Haptoglobin (Fig. 4d)		Iron (Fig. 4e)		Fibrinogen (Fig. 5a)		Thrombin-antithrombin (Fig. 5b)		D-dimer (Fig. 5c)	
	× 10^9^/L		g/L		mg/L		mg/L		μmol/L		g/L		μg/L		μg/mL	
Hours relative to LPS injection	Estimated mean	Range	Estimated mean	Range	Estimated mean	Range	Estimated mean	Range	Estimated mean	Range	Estimated mean	Range	Estimated mean	Range	Estimated mean	Range
−120	0.2	0.1–0.5	4.0	2–12	0.9	0.5–1.1	239.9	97.1–458.8	12.1	7.2–14.6	0.18	0.0–0.5	44.6	13.1–240.0	1.6	0.7–3.4
−96	1.6	0.1–11.6	11.3*	2–22	0.8	<DL-1.5	817.9*	207.2–5615.4	14.2	1.7–30.9	0.24	0.2–0.3	51.8	11.7–132.3	5.3	2.3–12.9
−24	0.6	0.2–2.0	6.5	2–12	<DL	<DL-14.2	284.2	108.1–670.3	16.3	6.1–31.5	0.35	0.0–0.6	24.8	11.2–57.7	1.4	0.0–6.8
0	2.2*	0.2–12.0	13.9***	6–30	0.5	<DL-1.1	600.0	128.9–2091.4	23.4	6.2–37.3	0.29	0.2–0.6	53.3	13.1–124.8	4.9	0.3–19.5
2	46.4***	17.1–100.3	30.6***	14–48	< DL	<DL-0.9	1166.3***	336.1–2391.3	23.7	14.0–33.8	0.60	0.3–1.0	720.0***	>720.0	6.3	0.7–30.0
4	111.2***	45.5–207.5	45.9***	38–64	<DL	<DL-0.9	1503.9***	531.7–2033.0	25.2	18.5–34.2	1.67***	1.2–2.1	720.0***	>720.0	2.0	0.8–3.1
8	159.8***	145.3–182	51.8***	46–64	1.3	0.9–1.7	1811.7***	1149.3–2231.3	21.9	5.0–37.2	2.07***	1.8–2.6	504.5***	286.9–720.0	2.1	1.7–2.7
16	93.6***	58.0–114.0	54.6***	50–64	23.7***	3.5–135.4	1846.0***	1468.4–2492.1	13.0	1.3–21.0	1.67***	1.2–2.3	249.4***	145.5–396.7	2.9	2.5–3.6
24	55.8***	31.2–89.8	48.1***	36–60	82.9***	19.8–294.7	1180.2***	897.8–1575.0	9.0	6.9–11.9	1.44***	1.2–1.6	186.3**	133.7–269.1	3.5	3.2–3.8
36	28.3***	14.8–47.5	44.7***	36–70	211.7***	45.6–905.8	1269.4***	435.3–3262.6	10.4	6.3–13.0	1.56***	1.1–1.8	151.7	81.2–234.7	6.2	3.3–17.4
48	22.1***	11.3–38.0	36.3***	29–46	231.2***	64.8–573.9	928.1**	373.8–1346.0	14.9	8.0–20.7	1.33***	0.6–1.7	193.5**	88.5–367.6	4.7	3.0–19.8
72	9.9***	3.5–48.0	31.8***	26–38	174.7***	50.6–470.1	619.2	307.9–991.5	17.2	13.3–25.3	1.07***	0.8–1.4	118.6	59.7–217.4	6.8	3.5–28.1
144	0.9	0.6–1.2	28.0***	20–40	2.1	0.2–29.1	696.1	528.0–1007.9	23.1	11.7–37.9	0.71	0.5–1.2	85.4	34.1–233.5	16.2***	9.6–20.9

*DL* Detection limit

*LPS* Lipopolysaccharide

Asterisks designate significant difference from post-injection hour −120 (* = *P* < 0.05; ** = *P* < 0.01 and *** = *P* < 0.001)

### Post-injection period: clinical parameters

The IA injection of LPS induced lameness starting at PIH 2, which resolved around PIH 48. Peak lameness scores were observed at PIH 2 and 4 (raw data are shown in Fig. 1a) with three horses being 5 out of 5 degrees lame at PIH 2 and/or 4. Pain scores peaked at PIH 4 (Fig. 1b; Table 2). Local inflammation was present with palpable heat and swelling from PIH 2 until the end of the study (data not shown). Pain reaction to palpation of the injected carpus was recorded between PIH 2 and 24 (data not shown). All horses showed increased heart rates, respiratory rates, and rectal temperatures (Figs. 1c-e; Table 2). Circumference and skin temperature of the injected carpus increased in all horses (Fig. 1f-g; Table 2).

### Post-injection period: biomarkers in synovial fluid

**Fig. 5:**
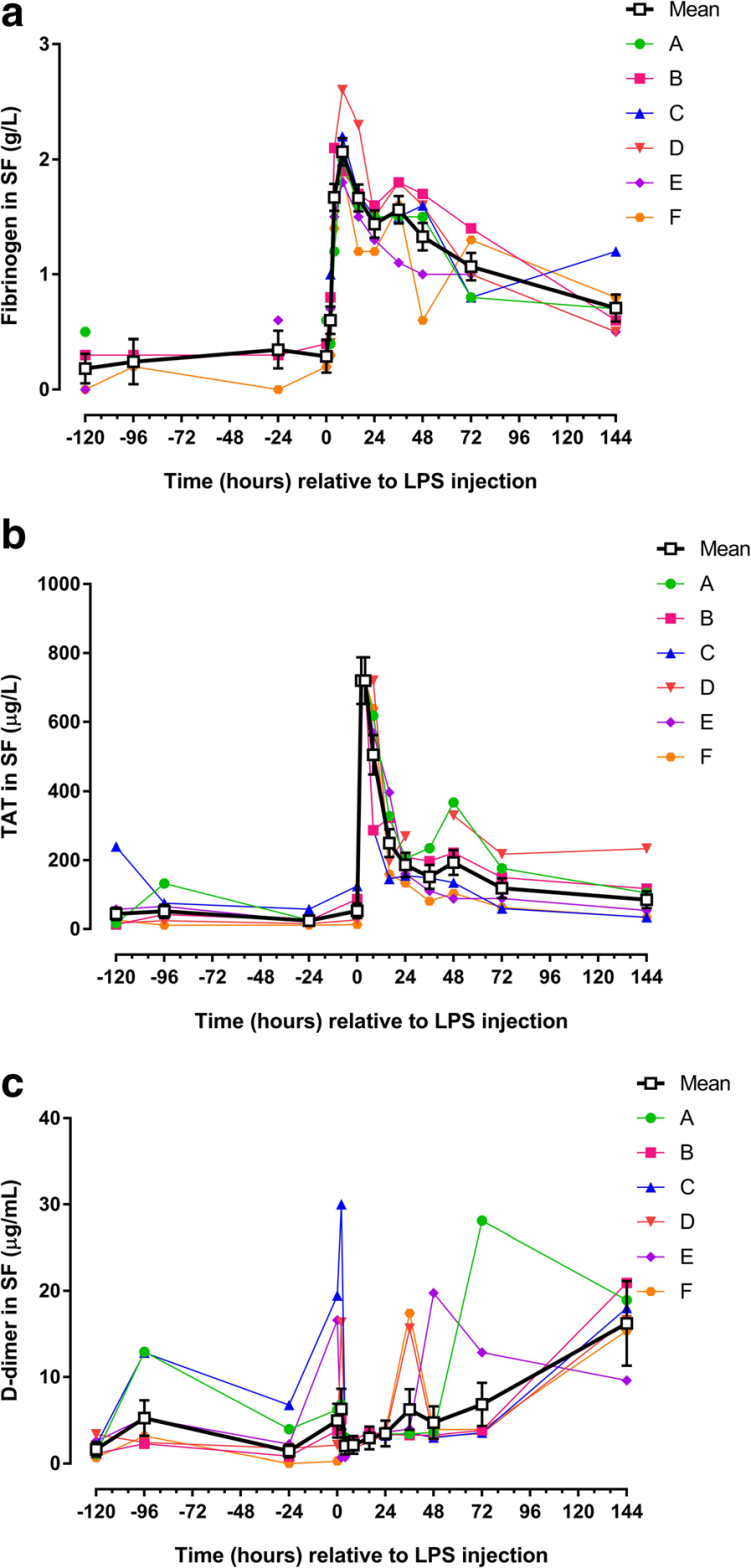
Concentrations/levels of haemostatic biomarkers measured in synovial fluid (SF) from 6 horses before and after intra-articular injection of 3 μg *E. coli* O55:B5 lipopolysaccharide (LPS) in one antebrachiocarpal joint (**a** fibrinogen; **b** thrombin-antithrombin [TAT]; **c** d-dimer). Individual horse responses and estimated means ± standard error of the mean *black line*) are depicted. Results of the statistical analyses are shown in Table 4

Visual inspection showed red or orange discolouration in 10 out of the 78 SF samples, thus suggesting that haemorrhage or haemolysis had occurred in the joint. Seven discoloured samples were from the pre-injection period, and 3 were from the post-injection period. Intra-articular injection of LPS caused statistically significant changes in all but one biomarker measured in SF. Five biomarkers showed rapid concentration changes with an early peak (WBC, TP, haptoglobin, fibrinogen and TAT [Figs. 4a-b,d and 5a-b; Table 4]). Concentrations of SAA increased more slowly (starting at PIH 16) (Fig. 4c; Table 4), as did d-dimer, concentrations of which were significantly increased only at PIH 144 (Fig. 5c; Table 4). While concentrations of most of the measured biomarkers returned to pre-injection levels before the end of the study, TP was increased for the entire duration of the study (Fig. 4B; Table 4). SF iron concentrations did not show a statistically significant concentration change after LPS injection (Fig. 4e; Table 4).

### Post-injection period: biomarkers in blood

Intra-articular injection of LPS caused significant changes concentrations of five out of eight parameters measured in blood. Four parameters increased significantly with peak values before PIH 48 (WBC, SAA, haptoglobin and fibrinogen [Figs. 2a,c-d, 3a; Table 3]); Iron concentration decreased significantly before PIH 24 (Fig. 2e; Table 3). Concentrations of TP and TAT appeared to increase with peak values within PIH 48, but changes were not statistically significant (Figs. 2b, 3b; Table 3). D-dimer concentration in blood did not change during the entire study period (Fig. 3c; Table 3).

## Discussion

The aim of the study was to characterize the IA haemostatic responses in experimentally-induced acute arthritis in horses and to relate it to IA inflammatory responses. Haemostatic and inflammatory responses in SF were activated quickly and simultaneously after IA LPS injection, which supports our hypothesis and their importance in equine inflammatory arthritis. The clinical signs and haematological and biochemical changes in blood and SF observed after IA injection of LPS were similar to those previously reported [30, 31]. While the horses developed pronounced systemic inflammation, concentrations of the haemostatic markers TAT and d-dimer did not change in blood after the IA injection of LPS, suggesting that spillage of inflammatory molecules such as cytokines occurs from the joint to the systemic circulation, whereas haemostatic responses remain confined to the joint compartment.

In equine joint research, focus has mainly been on inflammation and its adverse effects on joint tissues [6, 7]. In contrast, arthritis research in humans has for several years emphasized the importance of haemostasis in joint pathologies [10–13]. Haemostatic proteins such as TAT and fibrinogen have been detected in human SF, where they have been reported to induce or modulate inflammatory responses [14, 17]. Due to the low number of horses, no such assessment of interaction or of causal relationships between inflammatory and haemostatic biomarkers was made in this study.

### Haemostatic biomarkers

The increased concentration of fibrinogen in plasma observed after IA LPS injection corresponds to results from previous studies involving experimentally induced inflammation (arthritis, endometritis) in horses [36, 37], and it is a result of the acute phase response with increased hepatic synthesis of fibrinogen. The concentration of fibrinogen in SF started to increase after PIH 2 and reached an average maximum concentration of 2.1 g/L at PIH 8. Synovial fluid fibrinogen concentrations detected in the present study are in the same range as concentrations reported from human arthritis, where low levels of fibrinogen were found in healthy SF and concentrations up to 2 g/L were found in SF from patients with inflammatory joint diseases such as RA, gout and septic arthritis [19]. Accumulation of fibrinogen and fibrin in the joint compartment takes place in equine septic arthritis [38, 39] and may have deleterious effects on the tissues. Fibrin deposits are involved in the formation of pannus [40], a granulation tissue that traps bacteria and stimulates the release of cytokines, growth factors and other inflammatory biomolecules, thus causing cartilage destruction and bone erosion [41, 42]. Pannus formation in equine infected synovial structures has been related to reduced prognosis and poor athletic outcome after septic arthritis [38, 39]. The haemostatic functions of fibrinogen are well-known [43], but fibrinogen has also been shown to possess proinflammatory functions [16], as increased expression of inflammatory biomolecules were detected in cultured synoviocytes after fibrinogen stimulation [20, 21]. The balance between fibrin formation and dissolution is thought to be important for resolution of IA inflammatory disease [23, 24]. In the present study SF fibrinogen concentrations started to decrease after PIH 8. This decrease could be the result of fibrinolysis [25], and it could also be related to formation of fibrin that adhere to the synovial membrane and thus disappear from SF. Adherence of fibrin to the synovial membrane has been reported in mice 36 h after experimental induction of antigen-induced arthritis [18]. Adherence of fibrin was also observed arthroscopically 2 days after inoculation in an experimental *Staphylococcus aureus*-induced joint infection in horses [44]. Arthroscopic assessment or synovial membrane histology of horses with LPS-induced arthritis could provide more information on formation of IA fibrin deposits.

Our results showed that soluble fibrinogen can be measured in equine SF with the QuickVet® Equine Fibrinogen™ Test, but further studies are needed to fully explore the potential of fibrinogen as a biomarker of IA haemostatic pathways in equine inflammatory joint disease.

Intra-articular LPS injection induced a very fast and significant increase in SF TAT concentrations, but did not result in changes in plasma TAT concentrations. These results correspond to TAT concentrations demonstrated in plasma of healthy horses (2.6 ± 2 μg/L) [45] and in SF from humans with RA (1913.4 ± 1024 μg/L) [46]. A proteomic analysis of SF from horses with OA revealed a 2.2 fold increase in antithrombin level compared to SF from healthy animals [47]. In human inflammatory joint conditions, SF levels of TAT are correlated with SF tissue factor (TF) activity [11], which can induce thrombin generation [13]. Thrombin is rapidly inactivated in SF by antithrombin [13], and TAT can be considered an indicator of thrombin generation [48, 49]. In humans, TAT concentrations seemed to be related to degree of IA inflammation, as SF TAT concentrations were higher in RA than in OA (4430 ± 3580 μg/L versus 210 ± 260 μg/L) [14]. The results of the current study show that IA haemostasis was activated within 2 h after the inflammatory insult. The corresponding, rapid increase in SF fibrinogen, TP and WBC indicate that haemostasis was activated in synchronicity with inflammation in the joint compartment after IA LPS injection.

This is the first study to evaluate d-dimer concentration changes over time in acute equine joint inflammation. Plasma d-dimer concentration did not change in response to IA LPS injection, but in SF significantly increased d-dimer concentrations were demonstrated at PIH 144. Previous studies in horses have demonstrated increased d-dimer concentrations in SF from clinical cases of chronic joint disease with mild inflammation such as OA and OCD [26], as well as very high d-dimer concentrations in SF from foals with septic arthritis [25]. The increase in d-dimer concentration in inflammatory arthritis supports the hypothesis that inflammatory reactions in joint induce IA fibrinolysis [25]. D-dimer is thought to be produced locally in the joint [25], as also shown by SF d-dimer concentrations being much higher than those found in plasma in the present study. Factors involved in d-dimer generation (plasminogen, urokinase plasminogen activator and plasminogen activator inhibitor) have been demonstrated in SF [50, 51]. Since the joint inflammation induced in our model was of short duration and self-limiting, the present study cannot shed light on how fibrinolysis is activated in more complex joint diseases, but it seems that fibrinolysis occurs quite late in the course of joint inflammation. Conversely, the uniformly low level of d-dimer detected in all horses in the first 24 PIH could be indicative of an early decrease in fibrinolytic activity in the pathogenesis of joint diseases. This potentially very important balance between fibrinogenesis and fibrinolysis warrants further research.

### Inflammatory biomarkers

Measuring TP and WBC in SF is routine for assessment of joint inflammation [9]. Levels of WBC and TP in SF reached those reported to indicate presence of septic arthritis [5, 9], which confirms that the equine IA LPS model mimics this condition. The TP concentration in SF increased rapidly after IA LPS injection and reached concentrations close to those found in plasma. The majority of the SF protein content thus appeared to be derived from the systemic circulation (albumin).

Serum as well as SF SAA concentrations increased in response to IA injection of LPS. Increased SAA concentrations in SF as a result of IA inflammation have been demonstrated repeatedly in horses and other species [30, 31, 52–54]. A recent study suggested that septic arthritis results in particularly high SF SAA concentrations (> 100 mg/L) in horses [55], corresponding to levels found in our model of severe inflammatory arthritis. It has been shown that SAA is synthesized by IA tissues [56–58], and that SAA synthesis is elicited when cultured chondrocytes and fibroblast-like synoviocytes from horses are exposed to inflammatory molecules (e.g. LPS or proinflammatory cytokines) [59] or to molecules involved in haemostasis (thrombin, fibrinogen) [21].

Haptoglobin, a moderate acute phase protein in the horse [36], showed significantly increased serum concentrations from PIH 24 to the end of study. Previous studies have shown a serum haptoglobin response in horses with experimental arthritis [36], acute abdominal pain [60] and transportation stress [61]. Very little is known about the haptoglobin response in inflammatory joint disease in horses and other species [47, 51, 62], but a recent study demonstrated increased concentrations of haptoglobin in SF obtained from horses 15 days after experimental induction of arthritis by IA injection of amphotericin B [63]. In response to IA LPS injection, haptoglobin concentrations in SF peaked earlier than serum concentrations did (PIH 2–16). This very early peak makes it unlikely that IA de novo synthesis of haptoglobin had time to occur, and its presence in SF may be a result of blood contamination of SF. Haptoglobin may have protective effects in the joint, as it binds iron, thereby protecting against tissue degradation from oxidative damage [64, 65]. Haptoglobin has also been found to protect SF hyaluronic acid from free radical degradation [66]. There are no studies reporting the use of haptoglobin for diagnostic purposes in joint disease yet.

A decrease in iron concentration in serum was observed at PIH 16 and 24. This finding was expected, as it has been shown that LPS induce hepatic up-regulation of hepcidin, an iron metabolism regulator, which reduces iron availability in the blood stream [67] with proposed anti-microbial effects [68]. Effects of joint inflammation on SF iron concentration are not clear. Intra-articular LPS injection did not result in significant changes in SF iron concentrations in our horses, and previous studies in humans have shown opposing results, with one study showing higher SF iron concentrations in severe inflammation (RA) than in milder inflammation (OA) [69], while another study showed higher iron concentrations in SF from OA patients than in SF from RA patients and healthy individuals [70]. Intra-articular iron has been thought to be related to joint degradation processes through formation of free radicals [70, 71].

### Repeated arthrocenteses

The study design allowed us to assess the effect of repeated arthrocenteses on concentrations of SF biomarkers. Previous studies in calves and horses have shown that the trauma caused by insertion of a needle into the joint induces an IA inflammatory reaction with resultant significant changes in biomarker concentrations [32, 72, 73]. White blood cells counts, and TP and haptoglobin concentrations were increased in SF 24 h after the previous arthrocentesis, but 3 days after the previous arthrocentesis concentrations were back to pre-arthrocentesis levels. These findings are similar to those of Brama et al. [72], where the activity of matrix metalloproteinases in equine SF was increased 12 h after the first arthrocentesis and normalized after 72 h. Our group and others has previously shown that TP, but not concentrations of SAA or cartilage-derived retinoic acid-sensitive protein, are affected by previous arthrocentesis [30, 74, 75]. Synovial fluid WBC is increased 24–48 h after arthrocentesis [75, 76]. The IA response to arthrocenteses are thought to be caused by local inflammatory reactions in the synovial membrane [72], or by a minor haemorrhage with influx of cells and proteins that accumulates in SF [73, 77]. The latter seemed to occur in the present study as indicated by red or orange discolouration of 7 of the SF samples obtained in the pre-injection period. It is not clear if and how arthrocentesis-induced IA inflammation, haemorrhage or haemolysis may have affected measurements of the biomarkers in the present study. For some of the assays employed (e.g. TAT and d-dimer) the manufacturer specifically states that haemolysis does not affect measurement results. Bleeding, however, could potentially affect measured concentrations of biomarkers, either by proteins being delivered to SF by the haemorrhage (resulting in increased concentrations of the protein) or by blood plasma exerting a dilution effect (resulting in decreased protein concentrations).

Increased knowledge about effects of repeated arthrocenteses on concentration of SF biomarkers may be valuable for clinical diagnostic purposes.

## Conclusion

This study documented the concomitant induction of haemostatic and inflammatory responses in the joint compartment during experimental inflammatory arthritis in horses. The IA haemostatic responses occurred independently of the systemic haemostatic response and coincided with peak joint inflammation. The IA haemostatic response is elicited immediately after the inflammatory insult as evidenced by early increases in SF fibrinogen and TAT concentrations, while fibrinolysis of cross-linked fibrin with formation of d-dimer seemed to be activated late. These findings provide new insights into the pathogenesis of equine inflammatory arthritis. Future studies of the interaction between joint inflammation and haemostasis may prove important for development of new treatment modalities to improve the prognosis of severe joint inflammation.

## Abbreviations

EDTA: Ethylenediaminetetraacetic acid
IA: Intra-articular
LPS: Lipopolysaccharide
OA: Osteoarthritis
OCD: Osteochondritis dissecans
PIH: Post injection hour
RA: Rheumatoid arthritis
SAA: Serum amyloid A
SF: Synovial fluid
TAT: Thrombin-antithrombin
TF: Tissue factor
TP: Total protein
WBC: White blood cell count

## Footnotes

1 SF volume to 0.3 ml citrate = 2.7 ml blood x [100% - equine haematocrit of 45%].

2 SF volume to 4 ml spray-coated EDTA tube = 4 ml blood x [100% - equine haematocrit of 45%].
